# Induction of Hsp70 in tumor cells treated with inhibitors of the Hsp90 activity: A predictive marker and promising target for radiosensitization

**DOI:** 10.1371/journal.pone.0173640

**Published:** 2017-03-14

**Authors:** Vladimir A. Kudryavtsev, Anna V. Khokhlova, Vera A. Mosina, Elena I. Selivanova, Alexander E. Kabakov

**Affiliations:** Department of Radiation Biochemistry, A. Tsyb Medical Radiological Research Center, Obninsk, Russia; University of South Alabama Mitchell Cancer Institute, UNITED STATES

## Abstract

We studied a role of the inducible heat shock protein 70 (Hsp70) in cellular response to radiosensitizing treatments with inhibitors of the heat shock protein 90 (Hsp90) chaperone activity. Cell lines derived from solid tumors of different origin were treated with the Hsp90 inhibitors (17AAG, geldanamycin, radicicol, NVP-AUY922) or/and γ-photon radiation. For comparison, human cells of the non-cancerous origin were subjected to the same treatments. We found that the Hsp90 inhibitors yielded considerable radiosensitization only when they cause early and pronounced Hsp70 induction; moreover, a magnitude of radiosensitization was positively correlated with the level of Hsp70 induction. The quantification of Hsp70 levels in Hsp90 inhibitor-treated normal and cancer cells enabled to predict which of them will be susceptible to any Hsp90-inhibiting radiosensitizer as well as what concentrations of the inhibitors ensure the preferential cytotoxicity in the irradiated tumors without aggravating radiation damage to adjacent normal tissues. Importantly, the Hsp70 induction in the Hsp90 inhibitor-treated cancer cells appears to be their protective response that alleviates the tumor-sensitizing effects of the Hsp90 inactivation. Combination of the Hsp70-inducing inhibitors of Hsp90 with known inhibitors of the Hsp induction such as quercetin, triptolide, KNK437, NZ28 prevented up-regulation of Hsp70 in the cancer cells thereby increasing their post-radiation apoptotic/necrotic death and decreasing their post-radiation viability/clonogenicity. Similarly, co-treatment with the two inhibitors conferred the enhanced radiosensitization of proliferating rather than quiescent human vascular endothelial cells which may be used for suppressing the tumor-stimulated angiogenesis. Thus, the easily immunodetectable Hsp70 induction can be a useful marker for predicting effects of Hsp90-inhibiting radiosensitizers on tumors and normal tissues exposed to ionizing radiation. Moreover, targeting the Hsp70 induction in Hsp90 inhibitor-treated cancer cells and tumor vasculature cells may beneficially enhance the radiosensitizing effect.

## Introduction

In fight against cancer, radiotherapy is a powerful modality and often used for treating solid malignancies. However, there are two problems limiting application of radiotherapy and decreasing its efficacy: (1) many tumors are radioresistant, and (2) radiation exposure may cause severe damage to normal tissues. Both problems can be resolved or minimized by development of selective radiosensitizers which would be able to enhance the radiosensitivity of malignant cells without increasing the radiosensitivity of normal cells. In order to develop an appropriate radiosensitizer, it is necessary to perform preliminary research on identification of molecular targets responsible for radioresistance of cancer cells and also the focused screening of various agents interacting with those targets. In this respect, heat shock proteins, in particular, the 90 kDa and 70 kDa heat shock proteins (Hsp90 and Hsp70, respectively) seem to be the promising molecular targets for radiosensitization of tumors.

In eukaryotes, Hsp90 and Hsp70 are the major ATP-dependent cytosolic chaperones functioning as regulators of protein molecule conformations and protectors from cellular stresses [1,2]. Both chaperones are known to be involved in carcinogenesis, while their increased expression/activity in malignant cells is often correlated to the tumor progression, aggressiveness and resistance to therapeutics. In many model systems, inhibition of the expression or functional activity of these Hsps in tumors enabled to repress their malignant growth and sensitize them to the cytotoxic action of chemotherapeutic drugs or ionizing radiation [3–5]. That is why Hsp90 and Hsp70 are considered as very promising molecular targets for anticancer therapy and an active search of clinically applicable inhibitors of Hsps currently goes on.

Special attention is paid to Hsp90. Many client proteins of this chaperone (e.g. Raf-1, Akt, ATM, CDK4, HIF1α, ErbB2, BRCA1/2, survivin and others) are key components of signaling pathways responsible for unlimited proliferation of cancer cells, their resistance to apoptosis, repair of damaged DNA etc. Dysfunction of Hsp90 leads to inactivation and degradation of those client proteins, so that cell-permeable inhibitors of the Hsp90 activity can block multiple Hsp90-dependent reactions ensuring survival and proliferation of cancer cells [6]. Therefore, pharmacological inhibition of Hsp90 in patients’ tumors could directly exert the therapeutic effect and/or sensitize these tumors to conventional chemotherapy and radiotherapy. At present, a number of small molecule-based inhibitors of the Hsp90 activity are in preclinical testing or I-III phases of clinical trials as potential anticancer agents [7,8].

After experimental studies on various cell lines and tumor xenografts, several cell-permeable inhibitors of the Hsp90 activity were characterized as potent radiosensitizers of cancer cells, and perspectives of the application of analogous inhibitors in radiotherapy are discussed [9–11]. It was, however, found that the radiosensitizing effect of the Hsp90 inhibitors is not equally manifested in all types of malignancies: various tumors and cancer cell lines exist whose radioresistance was shown not to be impaired by the Hsp90-inhibiting treatments (reviewed in [9,10]). Furthermore, it was reported in some articles that the Hsp90 activity inhibitors such as geldanamycin, 17-N-allylamino-17-demethoxygeldanamycin (17AAG) or 17-dimethylaminoethylamino-17-demethoxygeldanamycin (17DMAG) radiosensitize malignant cells but not normal cells [12–15], whereas other authors asserted that human vascular endothelial cells can be radiosensitized with 17AAG [16].

Consequently, there is the need in an appropriate biomarker allowing to define (yet before radiation exposure) whether the treatment with Hsp90 inhibitors will result in the radiosensitization of target cells or not. One group of researchers who worked with 17DMAG (a water-soluble inhibitor of the Hsp90 activity) proposed to designate ErbB3 as such a biomarker: they asserted that if ErbB3 is expressed by tumor cells, these cells cannot be sensitized to radiation by means of Hsp90 inactivation [9,17]. However, it is not the universal determinant because there are ErbB3-negative tumor cell lines whose radiosensitivity is not enhanced by Hsp90 inhibitors. The genetic status (wild type or mutant) of the onco-suppressor protein p53 was also considered as a biomarker for similar goals: it was demonstrated on several cell lines of human oral squamous cell carcinoma that 17AAG (a hydrophobic inhibitor of the Hsp90 activity) increased the radiosensitivity of cells with the wild type of p53 rather than mutant [18]. In contrast, another report described 17AAG as a radiosensitizer of several human lymphoblastoid cell lines independently on their p53 status [19]. Thus, neither ErbB3 nor p53 seem to be an adequate marker for predicting the radiation response of Hsp90 inhibitor-treated cancer cells and there is a need to find a more suitable parameter.

An attempt to identify more universal and useful marker of the radiosensitization by Hsp90 inhibitors has been made in the present study. Here we propose to use, as the predictive marker, the Hsp70 induction that is the early, easily detectable cellular response to the Hsp90 dysfunction [20] and, according to our data, is causally associated with the radiosensitization of cells treated with Hsp90 activity inhibitors.

Likewise, based on the facts that inducible Hsp70 contributes to cellular radioresistance [21–23], we have supposed that the tumor-radiosensitizing action of Hsp90 activity inhibitors can be additionally enhanced by simultaneous prevention of the concomitant Hsp70 induction. In the present study, we tested this suggestion combining known inhibitors of the Hsp90 chaperone activity (geldanamycin, 17AAG, radicicol, NVP-AUY922) with inhibitors of the Hsp induction (quercetin [24], KNK437 [25], triptolide [26], NZ28 [27]). The data obtained demonstrate that Hsp70 induction in Hsp90 inhibitor-treated cells can serve as the predictive marker of the radiosensitization and, furthermore, it can be used as a molecular target for beneficial enhancing the radiation response in radioresistant tumors.

## Materials and methods

### Cell cultures

Cancer cell lines derived from human breast carcinomas (MCF-7, HBL-100), human uterine cervical carcinoma (HeLa), human prostate carcinomas (PC-3, Myc-CaP), human lung carcinoma (A549), human fibrosarcoma (HT 1080) and murine melanoma (B16) were obtained from the American Type Culture Collection (ATCC, Rockville, MD, USA). Two cell lines of human thyroid gland carcinomas FRO and KTC-1 were obtained from Dr. V.A. Saenko (Nagasaki University, Nagasaki, Japan). The *MDR1*-overexpressing MCF-7 breast cancer cell line (MCF-7/MDR1) and non-cancerous cell lines originated from human kidney epithelium (293) or human foreskin fibroblasts (BJ) were kindly provided by Dr. I.G. Kondrashova (Research Center for Molecular Diagnostics and Therapy, Moscow, Russia). All cell lines were routinely cultured under standard conditions (a humidified atmosphere with 5% CO_2_, 37°C) in complete growth medium (DMEM) supplemented with 10% fetal bovine serum, 2 mM L-glutamine and 1% penicillin/streptomycin.

The vascular endothelial cells were isolated from human umbilical veins and grown onto gelatin-coated substrates; the cells of two to six passages were maintained in endothelial growth culture medium-2 (EGM-2, Clonetics, East Rutherford, NJ) with supplements [16] and used for our experiments being in their pre-confluent (proliferating) or confluent (quiescent) state of the cell monolayer. To stimulate active proliferation in the cultured endothelium the pre-confluent cell monolayer was incubated in the growth medium containing 20% fetal bovine serum or recombinant human vascular endothelial growth factor (VEGF, Intergen, NY) and basic fibroblast growth factor (bFGF, Roche Molecular Biochemicals) at concentrations of 50 ng/mL for each [16].

### Compounds and treatments

Inhibitors of the Hsp90 activity [geldanamycin or radicicol (both from Biomol, Plymouth Meeting, PA) or 17AAG (Sigma, St. Louis, MO)], or NVP-AUY922 (Novartis, Basel, Switzerland) and inhibitors of the Hsp induction [quercetin (Sigma) or KNK437, or triptolide (both from Calbiochem, Darmstadt, Germany), or NZ28 (kindly provided by Dr. V.L. Gabai, Boston University Medical School, Boston, USA)] were dissolved in dimethyl sulfoxide (DMSO) and added to growth media over the cell cultures. In parallel, DMSO aliquots were introduced into control samples. Incubations of cells with the inhibitors were continued for 20–24 hours before radiation exposure.

Plastic Petri dishes with cell cultures were irradiated by γ-photons in a radiotherapeutic apparatus Luch-1 (Riga, Latvia) with ^60^Co at a dose rate of 1.3 Gy/min, so that cells received graded doses from 2 to 8 Gy [16,21].

The cell-stressing (non-lethal) hyperthermia was carried out by plunging hermetic Petri dishes with pre-confluent cell cultures into water in a thermostatic bath with temperature of 43°C for 1 hour or 45°C for 15 min.

### Clonogenic and MTT assays

After (co-)treatments, cells were trypsinized, harvested, counted, and then serially diluted and seeded in triplicate in 60-cm^2^ culture dishes. After 10–14 days, colonies (with a minimum of 50 cells) were fixed with ethanol, stained with 0.5% crystal violet and their number was counted. Relative clonogenicity was determined as the percentage of at least 50-cell-coloniesformed by the initially plated cells and normalized to the plating efficiency [28]. Dose-enhancement factors (DEFs) at a surviving fraction of 10% for several drug concentrations and combinations were calculated as described [16]. In addition, the mean survival fractions (SF) for each cell line were fitted to the linear quadratic model using Sigmaplot (Systat Software Inc.) and a known formula

SF = *exp(–α×D– β×D*^*2*^*)* where *D* is the irradiation dose and *α* and *β* are the fitted parameters.

The post-treatment cell viability was assessed in the MTT assay at different time periods following the cytotoxic treatments. The cells cultured in multi-well plates were incubated in the presence of 0.5 mg/ml MTT at 37°C for 3 h. Then the MTT-containing medium was removed, the cells were washed with phosphate buffer saline (PBS) and the *in situ* generated formazan product was solubilized by adding DMSO. Absorbance at 630 nm (background) was subtracted from absorbance at 570 nm for each well on a plate reader (Hewlett Packard) [16].

### Immunofluorescent staining

Cells grown on cover-slips were fixed with 3.5% formaldehyde and then permealized with 0.5% Triton X-100 in PBS. After the blocking step [1-hour-incubation in PBS with 1% bovine serum albumin (BSA) and 0.1% tween-20], the cell preparations were treated with a monoclonal antibody specifically recognizing an inducible form of Hsp70 (Rockland Immunochemicals, Gilbertsville, PA) or rabbit antibodies to phospho-histone γH2AX (Cell Signalling, MA, USA) diluted in the blocking buffer. Then, after triple washing with PBS, the cell preparations were stained with the respective secondary antibodies conjugated with Texas Red (anti-mouse Ig, Vector Laboratories) or Alexfluor 488 (anti-rabbit Ig, Invitrogen, Paisley, UK).

The fluorescence patterns were analyzed on a confocal microscope (Leica TCS SPE, Leica Microsystems, Heidelberg, Germany). The intracellular levels of inducible Hsp70 were quantified by measuring the average fluorescence intensity (brightness) per cell with the use of LAS software [29]. The nuclear γH2AX foci in the stained cell preparations were counted as recommended [30].

### Western blotting

Cells were lysed into Laemmli sample buffer containing a protease inhibitor cocktail; then aliquots of the freshly prepared lysates were run in sodium dodecyl sulfate-polyacrylamide gel electrophoresis and electrotransferred onto Hybond C membrane (Amersham, Arlington Heights, IL). After the blocking step [1-hour-incubation in PBS containing 5% fat-free dry milk], blots were probed with antibodies to an inducible form of Hsp70 (Enzo Life Sciences) or to phosphorylated HSF1 (Abcam) diluted in PBS with 1% BSA and 0.1% tween-20. Antibodies to β-actin (Sigma) were here used to equalize protein loading.

All the immunoreactive bands on blots were developed using peroxidase-conjugated secondary antibodies and the enhanced chemiluminescence (ECL) kit from Amersham. The developed bands of inducible Hsp70 and phosphorylated HSF1 were scanned and quantified using Kodak 1D Image Analysis software (Scientific Imaging Systems, Eastman Kodak Company, Rochester, NY, USA) and normalized to the β-actin levels.

### Luciferase refolding assay

The drug-induced inhibition of Hsp90 chaperone activity was monitored via delay of the Hsp90-dependent renaturation of luciferase in heat-stressed cells according to the reported technique [16]. A plasmid vector encoding cytoplasm-localized firefly luciferase under control of a Rous sarcoma virus [31] and Superfect reagent for transfection (Qiagen, Hilden, Germany) were used to transiently express the reporter enzyme in cell cultures studied. The luciferase-expressing cells were heated at 45°C for 15 minutes, then for recovery incubated at 37°C in the presence of cycloheximide to allow refolding of luciferase without its *de novo* synthesis. Before heating, graded concentrations of the Hsp90 inhibitors were added to the cells. At different time points after heating, cells were lysed by special ice-cold buffer, and then luciferase activity was measured in those lysates on a luminometer [16,31].

### Cell death determination

Cells in culture dishes were trypsinized and harvested 24 and 48 hours after irradiation and the cell suspensions were probed in double-label staining with fluorescein isothiocyanate (FITC)–conjugated annexin V and propidium iodide (PI) (both Calbiochem) as described [28]. Then the cell suspension samples were analyzed on a “FACS Vantage” cytometer (Becton Dickinson Immunochemistry Systems, USA). Being distinguished from still viable (unstained) cells, the fractions of apoptotic cells were identified as FITC-annexin V-positive but PI-negative cells, whereas the fractions of necrotic cells were PI-positive [28].

### Statistics

All quantitative data were calculated here as means ± SEM of four to eight separate experiments. Significance of differences between the sample groups and control was analyzed using ANOVA and additionally confirmed with the F-test.

## Results

### 1. Diversity in the Hsp70 expression in different cell cultures treated with the Hsp90 inhibitors

**Fig 1:**
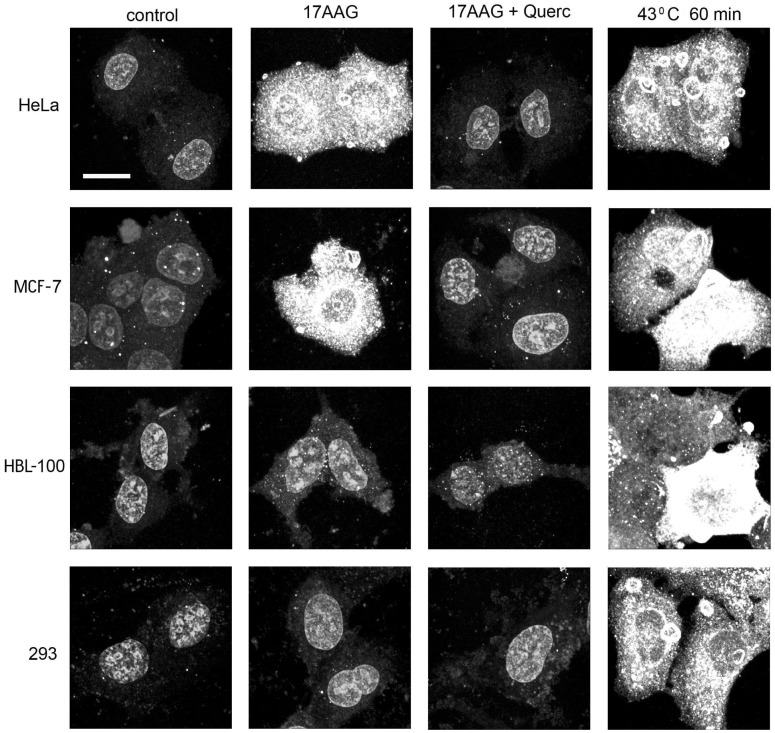
Texas-Red-immunofluorescence patterns of inducible Hsp70 in fixed/permeabilized cells of different cell lines. The cells were immunostained as untreated (control) samples or after 20 h incubation with 100 nM 17AAG without quercetin or in the presence of 40 μM quercetin, or 20 h after hyperthermia (43°C, 60 min); bar = 10 μm. It is clearly seen that the marked Hsp70 induction (the brightly stained cytoplasm) takes place in all cell samples exposed to hyperthermia as well as in HeLa cells and MCF-7 cells incubated with 17AAG, whereas 17AAG-treated HBL-100 cells and 293 cells are not stained. Very similar results were obtained with 50–200 nM geldanamycin or 30–100 nM radicicol, or 20–100 nM NVP-AUY922 instead of 17AAG and with 3–10 nM triptolide or 100–200 μM KNK437, or 5–20 μM NZ28 instead of quercetin (not shown). Similar variability in the expression of inducible Hsp70 was found in other cell cultures (Table 1).

**Fig 2:**
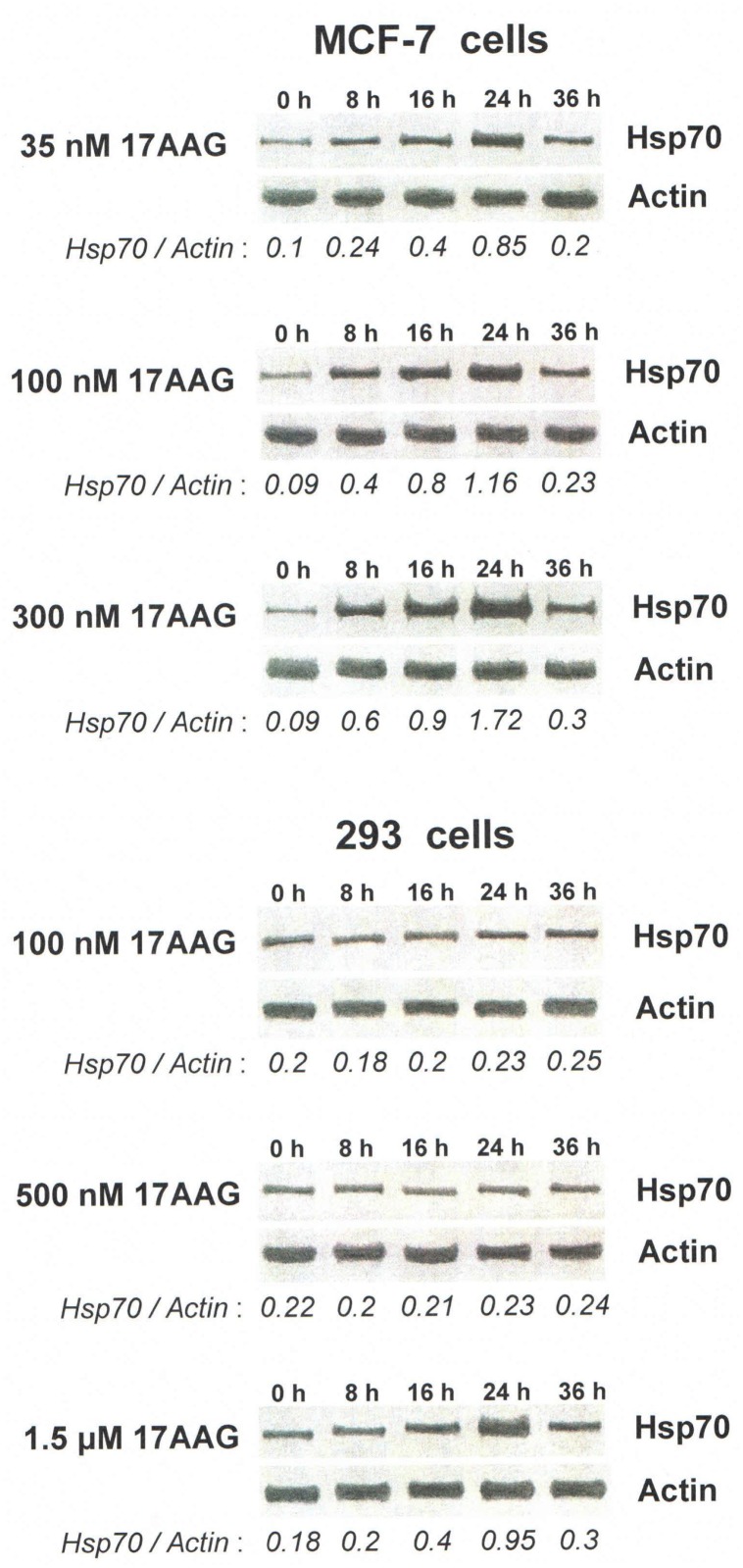
Western blots showing the diversity in dose-dependent effects of 17AAG on the Hsp70 induction in cancer MCF-7 cells and non-cancer 293 cells. The cells were lysed at different time points (indicated in hours along the upper sides of blots) of incubation with graded concentrations 17AAG and then analyzed with antibodies to inducible Hsp70 and β-Actin (load control). The values of Hsp70/Actin band ratio are presented along the lower sides of blots and reflect the relative amount of Hsp70 in cell samples. As it is seen, Hsp70 is induced in MCF-7 cells by much lower concentrations of 17AAG as compared to 293 cells. The similar difference was observed with other Hsp90 inhibitors in other cell cultures (see Table 1).

We found that dependent of concentrations, the Hsp90 inhibitors may exert diverse effects on cells of different origin. According to the data of immunofluorescent staining and immunoblotting, 35–400 nM (i.e. clinically achievable) concentrations of 17AAG, NVP-AUY922, geldanamycin and radicicol caused a prominent induction of Hsp70 in the inhibitor-treated cancer cells such as MCF-7, HeLa, KTC-1, PC-3, Myc-CaP, A549 and HT 1080. In these cells, the average intracellular levels of an inducible form of Hsp70 increased 4-6-fold after 16–24 hours in a dose-dependent manner as compared with the basal levels of the constitutive expression of the protein in untreated (control) cells (Figs 1 and 2, Table 1). The 3–3.5-fold increase in the levels of inducible Hsp70 can also be detected in those cells already at the earlier time points in 6–8 hours since the start of the inhibitory treatment (Fig 2).

**Table 1:** Comparative data on the expression of inducible Hsp70 in cancer and non-cancer cell cultures treated with hyperthermia or inhibitors of the Hsp90 activity.

Cell Culture	Untreated control	43°C for 60 min	17AAG 50 nM	17AAG 100 nM	Geldanamycin 100 nM	Radicicol 50 nM	NVP-AUY92250 nM
**HeLa**	6 ± 0.5	20 ± 2.1*	23 ± 2.4*	32 ± 3.4*	40 ± 4.2*	28 ± 3.0*	37 ± 3.75*
**MCF-7**	4.5 ± 0.4	23 ± 2.5*	14.2 ± 1.6*	22 ± 2.4*	27 ± 3.0*	24.5 ± 2.5*	28 ± 2.9*
**MCF-7/MDR1**	5.0 ± 0.3	24 ± 3.0*	8.5 ± 0.7**	13 ± 1.2**	14.5 ± 1.3**	14 ± 1.35**	15.6 ± 1.6**
**HBL-100**	4.0 ± 0.35	22 ± 2.3*	4.8 ± 0.5	6.4 ± 0.7	6.8 ± 0.65	6.5 ± 0.6	6.7 ± 0.7
**KTC-1**	5.5 ± 0.5	21 ± 2.0*	18 ± 2.1*	27 ± 2.8*	28.2 ± 0.3*	29 ± 3.1*	30 ± 0.3*
**FRO**	4.8 ± 0.4	22 ± 2.1*	5.0 ± 0.5	5.2 ± 0.5	4.9 ± 0.45	5.1 ± 0.5	5.3 ± 0.55
**PC-3**	5.6 ± 0.6	24 ± 2.5*	17 ± 1.8*	25 ± 2.65*	24 ± 2.6*	26 ± 2.5*	28 ± 2.9*
**Myc-CaP**	5.3 ± 0.55	25 ± 2.7*	16 ± 1.7*	24 ± 2.6*	25 ± 2.7*	25 ± 2.8*	27 ± 2.8*
**A549**	5.7 ± 0.6	16 ± 1.4*	13 ± 1.5*	18 ± 1.9*	19 ± 1.85*	17.5 ± 1.6*	20 ± 2.1*
**HT 1080**	6 ± 0.65	23 ± 2.1*	18 ± 2.0*	26 ± 2.5*	25 ± 2.3*	25.2 ± 2.7*	29 ± 3.0*
**B16**	3.6 ± 0.4	16.7 ± 0.4*	3.8 ± 0.35	4 ± 0.4	3.7 ± 0.3	4 ± 0.38	3.8 ± 0.35
**293**	8.7 ± 0.7	27 ± 0.3*	9.18 ± 0.9	10.5 ± 1.0	11 ± 1.0	10.4 ± 1.0	10 ± 1.1
**BJ**	6.9 ± 0.65	25 ± 2.7*	7.2 ± 0.8	7.5 ± 0.7	7.8 ± 0.8	7.6 ± 0.75	7.7 ± 0.8
**Proliferating EC**	6.5 ± 0.7	24 ± 2.5*	17 ± 1.6*	21 ± 0.22*	20 ± 0.2*	22 ±0.21*	22 ± 0.23*
**Quiescent EC**	6 ± 0.65	25 ± 2.6*	7.5 ± 0.8	8.3 ± 0.85	8.5 ± 0.9	8.2 ± 0.75	8.5 ± 0.8

*Notes to Table 1*. These data are average values of the Texas-Red-fluorescence brightness per cell that reflect the expression levels of inducible Hsp70 in various cells immunostained with the specific anti-Hsp70 antibody 20 h after either treatment (see Fig 1). Each value is the mean ± SEM from results of 4 independent experiments.

*—significant differences from respective control values (p < 0.05).

However, nanomolar (35–500 nM) concentrations of the Hsp90 inhibitors did not cause the Hsp70 induction in the other cancer cell lines: HBL-100, FRO and B16 (Fig 1). In MCF-7/MDR1 cells, in which the intracellular accumulation of all Hsp90 inhibitors was suppressed owing to the overexpression (and hyperactivity) of transmembrane P-glycoprotein working as a drug-excluding pump [32], the level of inducible Hsp70 was significantly lower than in wild type MCF-7 cells subjected to the same inhibitory treatment (Table 1).

As for the non-cancer cell lines (293 and BJ), no marked increase in the inducible Hsp70 expression occurred in them after treatments with 35–500 nM concentrations of the Hsp90 inhibitors (Figs 1 and 2, Table 1). Studies of the human vascular endothelium revealed the considerable diversity in the actively proliferating and quiescent cell cultures: in the low density cultures of proliferating (serum- or growth factor-stimulated) endothelial cells, strong Hsp70 induction in response to the treatments with nanomolar concentrations of the Hsp90 inhibitors was always observed. In contrast, the tight monolayer of quiescent (non-proliferating) endothelial cells exhibited no significant induction of Hsp70 following the same inhibitory treatment (Table 1).

It should be noted that in contrast to the Hsp90-inhibiting drug treatments, mild hyperthermia (43°C, 30 min), being used here as a positive control for Hsp-inducing stimuli, caused virtually equal induction of Hsp70 in all the cell cultures examined (Fig 1, Table 1).

### 2. Radiosensitizing effects of the Hsp90 inhibitors correlate with the level of Hsp70 induction in target cells

**Fig 3:**
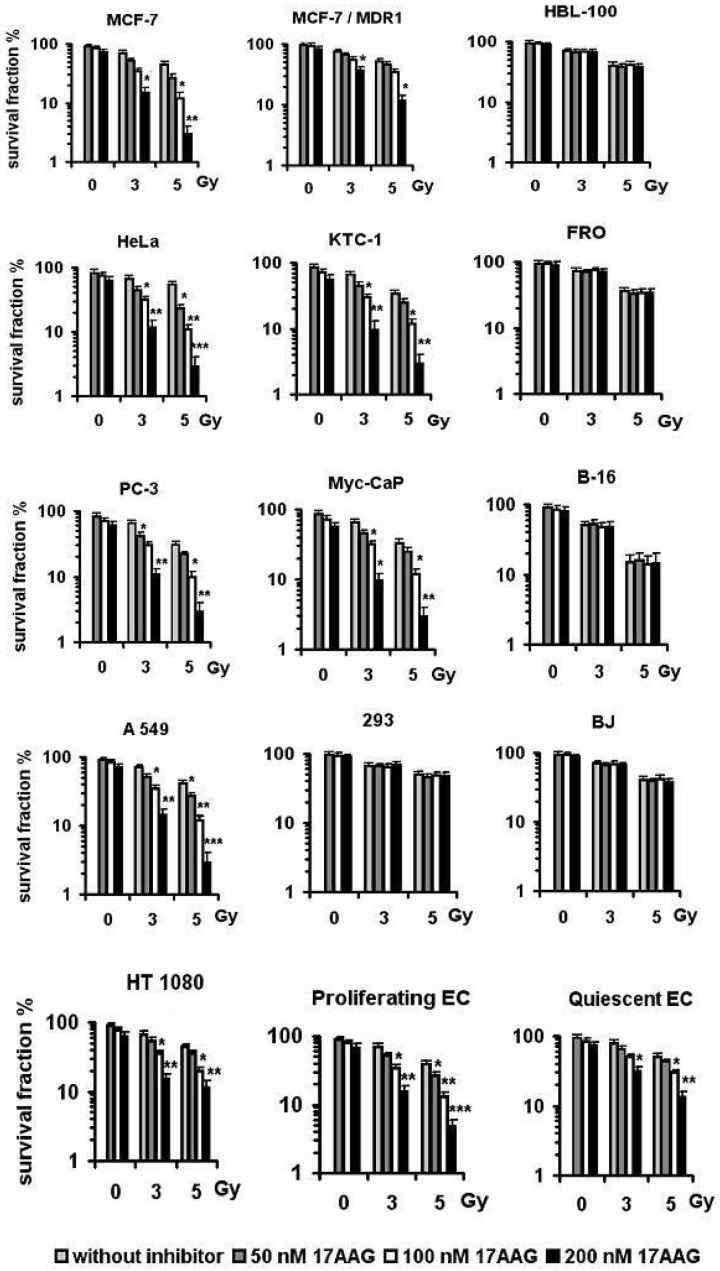
The diversity in effects of 17AAG on the post-radiation clonogenicity of various cell cultures. The cells were incubated with various concentrations of the Hsp90 inhibitor for 24 h before γ-irradiation at clinically relevant doses. Here it is demonstrated the drug-conferred radiosensitization of HeLa, MCF-7, KTC-1, PC-3, Myc-CaP, A549 and HT 1080 cancer cells and also of actively proliferating (pre-confluent) endothelial cells, whereas no radiosensitizing effect takes place on HBL-100, FRO and B16 cancer cell lines, non-cancerous 293 and BJ cell lines and quiescent (tight monolayer) endothelial cells. The presented bars express mean ± SEM of 4–5 independent experiments. *—significant difference from the neighboring unmarked bars, p<0.05; **—significant difference from the unmarked bars and bars marked with *, p<0.05. ***—significant difference from all the neighboring bars in each group of samples, p<0.01.

During studying effects of the Hsp90 inhibitors (17AAG, NVP-AUY922, geldanamycin and radicicol) on the cellular radiation response, we found that 35–500 nM of the drugs significantly increased the radiosensitivity of MCF-7, HeLa, KTC-1, PC-3, Myc-CaP, HT 1080 and A549 cancer cell lines as well as actively proliferating endothelial cells, i.e. only those cell cultures in which the prominent Hsp70 induction was found after the inhibitory treatment. On the contrary, no substantial radiosensitization occurred in HBL-100, FRO and B16 cancer cell lines, non-cancerous 293 and BJ cell lines and quiescent (confluent) endothelial cell monolayer treated in the same way (see Fig 3 and Table 2). It seems interesting that the carcinoma cell lines derived from the same type of human tissues (e.g. MCF-7 and HBL-100 –from the mammary gland; KTC-1 and FRO—from the thyroid gland) can exhibit absolutely different responses to the (co-)treatments with the Hsp90 inhibitors.

**Table 2:** The enhanced radiosensitization of cancer cells and proliferating endothelial cells (pr EC) by combinations of inhibitors of the Hsp90 activity (17AAG, NVP-AUY922) and inhibitors of the Hsp70 induction (quercertin, triptolide).

Cells	Radiosensitizing treatments (1) and dose enhancement factors (2)		
**HeLa**	**1.** 100 nM 17AAG	**1.** 80 nM 17AAG + 40 μM Querc	**1.** 80 nM 17AAG + 5 nM Tript
	**2.** 1.65	**2.** 2.54	**2**. 2.64
	**1.** 50 nM NVP	**1.** 50 nM NVP + 40 μM Querc	**1.** 50 nM NVP + 5 nM Tript
	**2**. 1.7	**2**. 2.62	**2**. 2.71
**MCF-7**	**1.** 100 nM 17AAG	**1.** 80 nM 17AAG + 40 μM Querc	**1.** 80 nM 17AAG + 3 nM Tript
	**2**. 1.47	**2**. 2.15	**2**. 2.32
	**1.** 50 nM NVP	**1.** 50 nM NVP + 40 μM Querc	**1.** 50 nM NVP + 3 nM Tript
	**2**. 1.63	**2**. 2.43	**2**. 2.56
**KTC-1**	**1.** 100 nM 17AAG	**1.** 80 nM 17AAG + 40 μM Querc	**1.** 80 nM 17AAG + 5 nM Tript
	**2**. 1.54	**2**. 2.26	**2**. 2.4
**PC-3**	**1.** 100 nM 17AAG	**1.** 80 nM 17AAG + 40 μM Querc	**1.** 80 nM 17AAG + 5 nM Tript
	**2**. 1.49	**2**. 2.5	**2**. 2.42
	**1.** 50 nM NVP	**1.** 50 nM NVP + 40 μM Querc	**1.** 50 nM NVP + 5 nM Tript
	**2**. 1.55	**2**. 2.71	**2**. 2.49
**pr EC**	**1.** 100 nM 17AAG	**1.** 80 nM 17AAG + 40 μM Querc	**1.** 80 nM 17AAG + 4 nM Tript
	**2**. 1.56	**2**. 2.2	**2**. 2.28

*Notes*. The DEF values were calculated for the 10% post-radiation cell colony survival and certain concentrations of 17AAG, NVP-AUY922 (NVP), triptolide (Tript) and quercetin (Querc) which exerted no or minimal cytotoxic effects without subsequent γ-irradiation (2–8 Gy), while the Hsp-inhibiting activities were still manifested.

Importantly, the concentration thresholds of 17AAG, NVP-AUY922, geldanamycin and radicicol under which the Hsp70 induction became well-detectable in the treated cells fully coincided with those which began to confer the radiosensitization. Exactly the same concentrations of the Hsp90 inhibitors which maximally stimulated the Hsp70 expression usually exerted the strongest radiosensitizing effects (Figs 2 and 3).

Comparison of the effects in MCF-7 and MCF-7/MDR1 lines also revealed good correlation between, the level of Hsp70 induction and degree of the radiosensitization: after the same inhibitory treatments, both the Hsp70 induction and the achieved radiosensitization were significantly weaker in MCF-7/MDR1 cells actively excluding the drugs than in MCF-7 cells (see Fig 3 and Table 2).

The same tendency was found when comparing (i) actively proliferating and (ii) quiescent cell cultures of the human vascular endothelium: the former exhibited the strong Hsp70 induction and a fairly high degree of the radiosensitization by Hsp90 inhibitors, whereas the latter, being equally treated, responded by the weaker induction of Hsp70 (Table 1) and only slight enhancement of their radiosensitivity (Fig 3, Table 2). Therefore, the Hsp90 activity inhibition may be used for the selective radiosensitization of the vascular endothelium involved in tumor-stimulated angiogenesis, as it was previously suggested [16].

Taking into consideration that 17AAG and related Hsp90-binding agents can cause activation of the heat shock transcription factor-1 (HSF1) with subsequent HSF1-mediated induction of other Hsps besides Hsp70 [20], we examined whether other Hsps become up-regulated like Hsp70 in the Hsp90 inhibitor-treated cancer cells. In our model with selected cancer cell lines (MCF-7, HeLa, KTC-1, PC-3, Myc-CaP and HT 1080) and proliferating endothelial cell cultures, nanomolar concentrations of 17AAG, NVP-AUY922, geldanamycin and radicicol yielded only a slight (20–30%) increase in the intracellular levels of Hsp27 and Hsp40 but no significant changes in the expression of Hsp110, Hsp90 and Hsp60 (data not shown). On the contrary, the dramatic (several-fold) up-regulation in the levels of inducible Hsp70 over the basal levels its constitutive expression was always observed in those cells after such inhibitory treatments (see Figs 1 and 2, Table 1). Thus, among the major inducible Hsps, it is Hsp70 that seems to be a cellular marker of the Hsp90 dysfunction and the Hsp90 inhibitor-conferred radiosensitization.

### 3. Targeting Hsp70 induction in Hsp90 inhibitor-treated cells can enhance the radiosensitizing effect

While the drug-induced Hsp90 dysfunction promotes the radiosensitization of cancer cells, the simultaneously occurring induction of Hsp70 may contribute to their radioprotection because up-regulated Hsp70 makes cells more radioresistant [21–23]. The very similar situation may happen with endothelial cells of the tumor vasculature treated with Hsp90-inhibiting drugs and then irradiated. Thus, up-regulation of Hsp70 may attenuate radiosensitization conferred by Hsp90 inhibitors. To solve this problem we propose to use the Hsp90 activity inhibitors in combination with inhibitors of the Hsp70 induction: this enables to enhance the tumor cell radiosensitization by blocking the undesirable up-regulation of radioprotective Hsp70.

**Fig 4:**
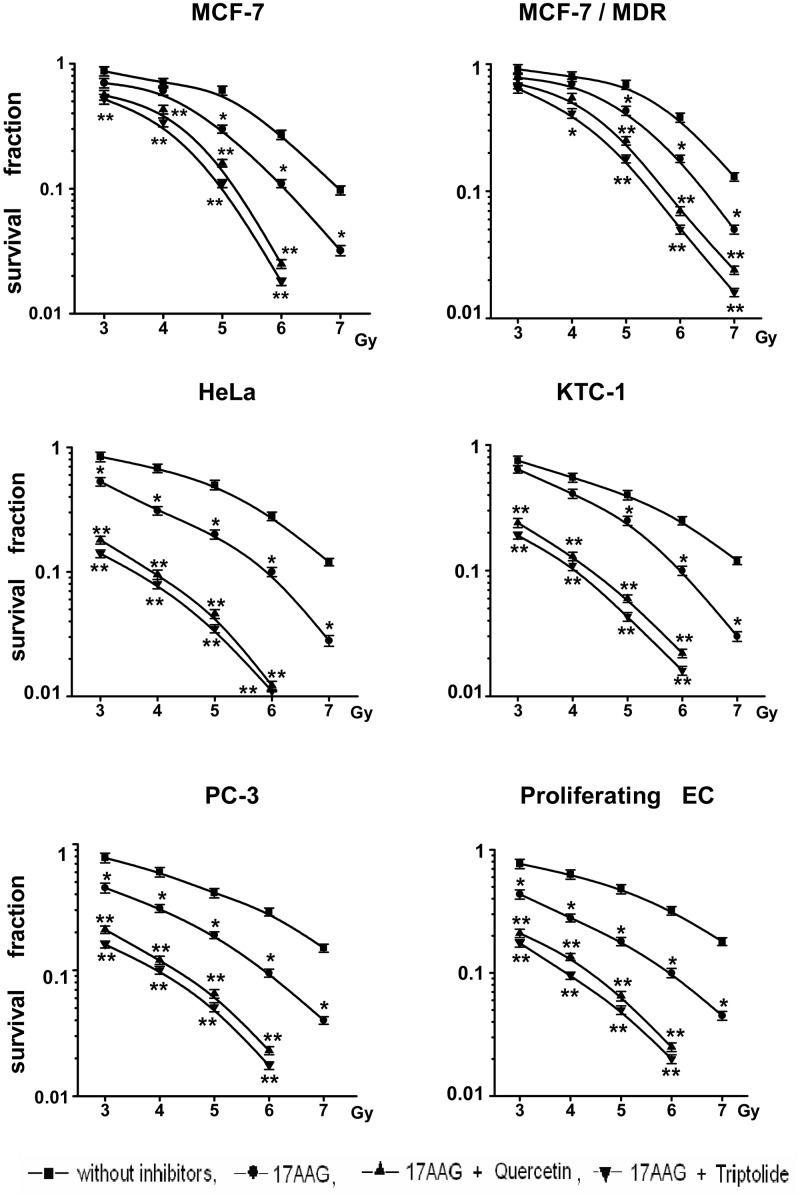
The enhanced radiosensitization of certain cell cultures with combination of 17AAG with inhibitors of the Hsp70 induction. The curves of post-radiation survival of colony-forming cells show the considerable decrease in the survival fractions when the up-regulation of inducible Hsp70 in 17AAG-treated cells was blocked by quercetin (Querc) or triptolide. The similarly enhanced radiosensitization was also found in other cell cultures (A549, HT 1080 and Myc-CaP) with 50–200 nM geldanamycin or 30–100 nM radicicol, or 50–200 nM NVP-AUY922 instead of 17AAG and with 100–200 μM KNK437, or 5–20 μM NZ28 instead of quercetin and triptolide (see also Table 2). *—significant difference from control, p<0.05. **—significant difference from control and values marked with *, p<0.05.

**Fig 5:**
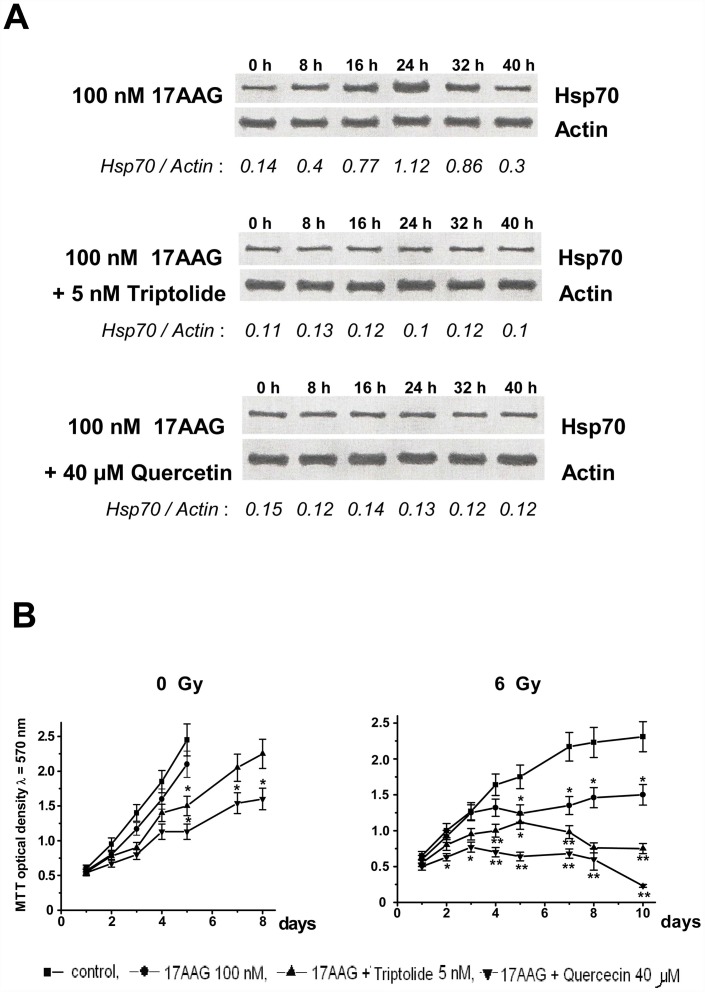
The data of Western blotting and MTT assay demonstrating the enhanced radiosensitization of 17AAG-treated HeLa cells by preventing the Hsp70 induction with triptolide or quercetin. The presented blots (**A**) demonstrate that both inhibitors of the Hsp induction completely abrogated up-regulation of inducible Hsp70 in response to the 17AAG treatment. (The values of Hsp70/Actin band ratio are presented along the lower sides of blots and reflect the relative amount of Hsp70 in cell samples. Of note, such co-treatments with the Hsp70 induction inhibitors did not decrease the basal level of constitutively expressed Hsp70 in target cells). The presented curves (**B**) show that in contrast to the action of 17AAG alone, the two-inhibitor combinations prevented the post-radiation recovery of proliferative activity in the drug-treated cells. Very similar results were obtained with 50–200 nM geldanamycin or 30–100 nM radicicol, or 50–200 nM NVP-AUY922 instead of 17AAG and with 100–200 μM KNK437, or 5–20 μM NZ28 instead of quercetin and triptolide (not shown). MTT assay also revealed the enhanced radiosensitization of MCF-7, KTC-1, PC-3, Myc-CaP and HT 1080 cancer cells pretreated with combination of the Hsp90 activity inhibitors and inhibitors of the Hsp70 induction (not shown). The presented data express mean ± SEM of 5 independent experiments. *—significant difference from the respective unmarked values, p<0.05; **—significant difference from the respective unmarked or marked with * values, p<0.05.

The data of both clonogenic (Fig 4) and MTT (Fig 5) assays demonstrate significantly stronger cancer cell radiosensitization in the case of combining the Hsp90 activity inhibitors with the Hsp induction inhibitors such as quercetin, KNK437, triptolide or NZ28. This phenomenon of the enhanced radiosensitization took place only in the cell cultures sensitive to the action of Hsp90 activity inhibitors, namely in MCF-7, HeLa, KTC-1, PC-3, Myc-CaP, HT 1080 and A549 cancer cell lines and actively proliferating endothelial cells but not in HBL-100, FRO and B16 or quiescent endothelial cells. Even the radiosensitivity of MCF-7/MDR1 was somewhat increased by means of such combinative co-treatments with the two inhibitors (Fig 4). All the values of DEFs calculated for several cancer cell lines were significantly higher under the combination *Hsp90 activity inhibitor + Hsp70 induction inhibitor* than those following the action of Hsp90 inhibitor alone (some values of the DEFs are given in the Table 2). When quercetin, KNK437, triptolide or NZ28 were added in lower concentrations which do not suppress the Hsp70 induction in the Hsp90 inhibitor-treated cancer cells, no enhanced radiosensitization was observed (data not shown).

In all performed experiments, the enhancement of radiosensitization following the double-inhibitor-treatment was never found in normal (non-cancerous) human cells, except actively proliferating endothelial cells (see below), that well correlated with the difference in their expression of inducible Hsp70 (see Figs 1 and 2, Tables 1 and 2). Such selectivity in the radiosensitization of cancer cell cultures allows to hope that, in the case of *in vivo* application of this method, the irradiation-associated cytotoxicity will preferentially be enhanced within the tumor volume without aggravating the radiation damage to adjacent normal tissues.

The double-drug combination *Hsp90 activity inhibitor + Hsp70 induction inhibitor* considerably enhanced the radiosensitization of actively proliferating vascular endothelial cells as compared with the effect of Hsp90 activity inhibition alone (Fig 4, Table 2). Such radiosensitizing effects did not take place in the quiescent endothelial cells exposed to the same co-treatments (not shown); this difference appears to be due to the different responses to the Hsp90 activity inhibitors in both endothelial cell cultures explored (see Table 1). If analogous approach is applicable *in vivo*, this may help to suppress the tumor-stimulated angiogenesis without excessive injury of normal blood vessels located at the way of γ-photon rays.

### 4. A possible mechanism of the observed phenomena

**Fig 6:**
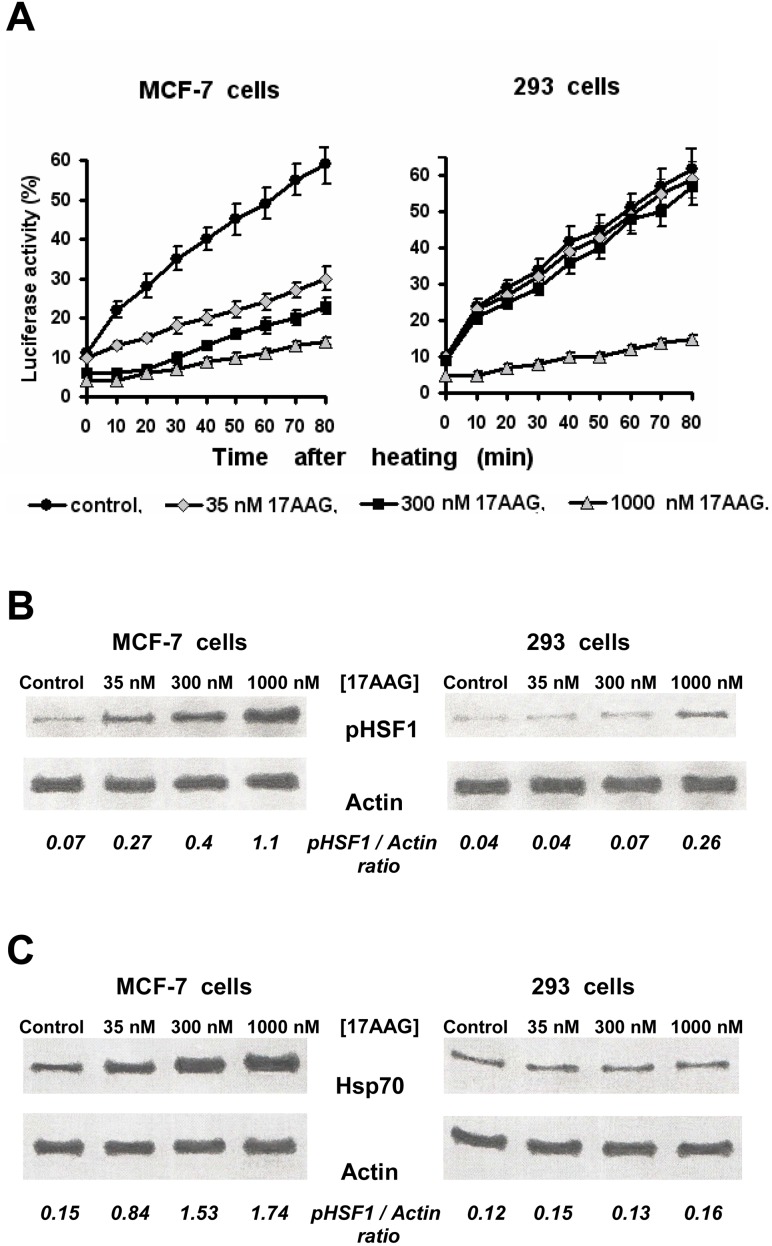
Various cell cultures can exhibit the different susceptibility of their Hsp90 chaperone machine and their HSF1-mediated Hsp70 induction to inhibitors of the Hsp90 activity. Here it is seen that much lower concentrations of 17AAG are required to repress the Hsp90 chaperone function-dependent refolding of luciferase (**A**) and stimulate the HSF1 phosphorylation (**B**) and the Hsp70 induction (**C**) in MCF-7 breast cancer cells as compared with non-cancerous 293 cells. Numbers under the blots represent the expression of phosphorylated HSF1 (pHSF1) or inducible Hsp70 relative to β-actin. Both cell cultures were treated with 17AAG for 20 h before analyses.

The phenomenon of induction of Hsp70 in mammalian cells treated with inhibitors of the Hsp90 activity is well known and explained by the HSF1 activation in response to the drug-provoked chaperone dysfunction [20]. Hence, we tried to reveal at the molecular level a relationship between the selectivity in the action of Hsp90 activity inhibitors and a degree of the chaperone dysfunction in different cell cultures subjected to the inhibitory treatment. In the assay with Hsp90-dependent refolding of heat-denatured luciferase, we indeed found that different concentrations of the Hsp90 inhibitors (17AAG, NVP-AUY922, geldanamycin and radicicol) cause a diverse degree of the chaperone dysfunction in the treated cells: nanomolar concentrations of the Hsp90 inhibitors substantially delayed the post-stress luciferase refolding only in cell lines where the prominent Hsp70 induction took place following the same inhibitory treatments, i.e. in HeLa, KTC-1, PC-3, Myc-CaP, HT 1080 and MCF-7 cancer cells (see Fig 6A for example) and also in actively proliferating endothelial cells. Importantly, the same (nanomolar) concentrations of the Hsp90 inhibitors resulted in the HSF1 activation (phosphorylation) in these cells, in contrast to the other cell cultures which are non-susceptive to the Hsp90 inhibitors (Fig 6B). Thus, the Hsp70 induction does not occurs if the cellular chaperone machinery is not inactivated by treatments with the Hsp90 inhibitors. These data are in full accord with the data shown in Fig 2. The similar difference was observed comparing the effects of the Hsp90 inhibitors on HeLa, KTC-1, PC-3, Myc-CaP and HT 1080 cells with those on HBL-100, FRO and B16 cells (not shown).

Furthermore, we tried to investigate why some cell cultures remain insensitive to nanomolar concentrations of the Hsp90 inhibitors, whereas others react to them quite well. Taking into consideration the observed diversity in the effects of the Hsp90 inhibitors on MCF-7 cells and MCF-7/MDR1 cells (see Fig 3, Table 1), the simplest explanation could be that the “insensitive” cell cultures have high levels of MDR1 product (P-glycoprotein) expression/activity allowing them to exclude Hsp90 inhibitors. However, co-treatments with verapamil (a known blocker of the drug-pumping-out activity of P-glycoprotein [32]), while it sensitized MCF-7/MDR1 cells to 17AAG and geldanamycin, did not exert analogous effects toward HBL-100, FRO and B16 cancer cells (not shown). Probably, there is another (unrelated to MDR1) reason for the relative resistance of those cancer cell lines to Hsp90 activity inhibitors.

**Fig 7:**
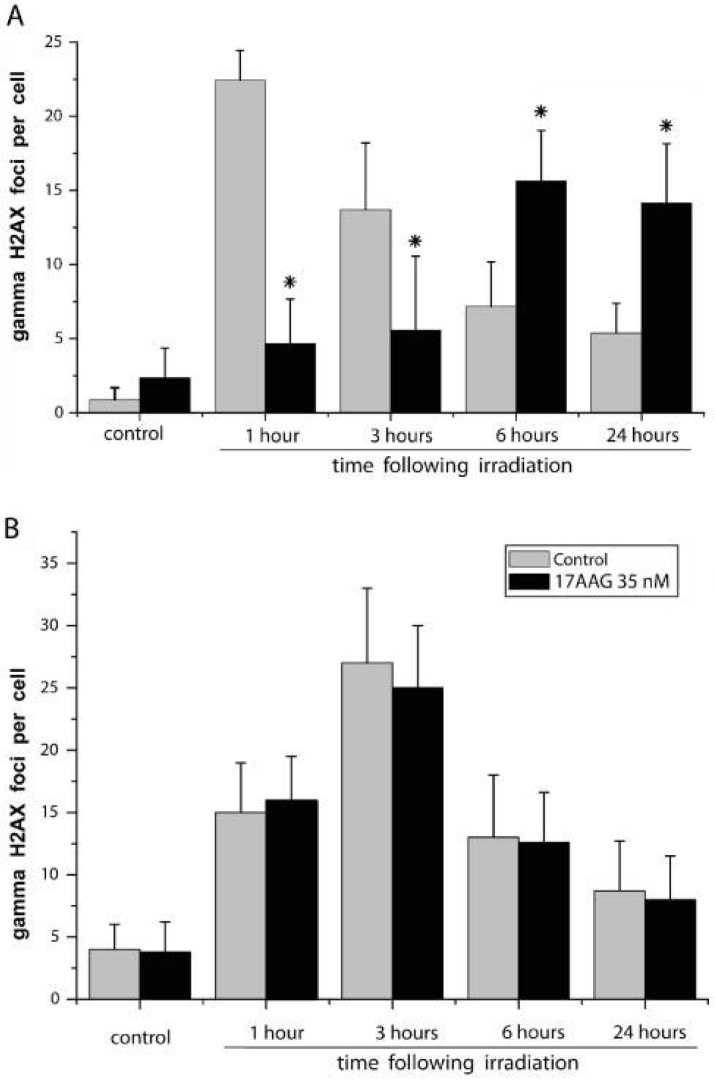
The diversity in dynamics of appearance and disappearance of the γH2AX foci in nuclei of non-cancerous 293 cells and MCF-7 breast cancer cells pretreated with 17AAG before radiation exposure. The presented bars show average amounts (mean ± SEM) of the γH2AX foci per cell nucleus at different time points following irradiation (4 Gy). **A**–MCF-7 cells: at all time points following irradiation, the effects of 17AAG (marked by *) significantly differ from respective values in control, p<0.05. **B**– 293 cells: no significant differences between the 17AAG-treated cells and control.

The next point in our studies was elucidation of the mechanism of selective radiosensitization conferred by the Hsp90 inhibitors. It was previously suggested by other researchers [15,33–37] that inhibition of the Hsp90 activity compromises post-radiation DNA repair in irradiated human tumor cells. To test this suggestion in our model, we measured amounts and dynamics of the double-strand breaks in nuclear DNA of cells (co-)treated with the Hsp90 inhibitors and/or radiation. In fact, we found that pretreatments of MCF-7 cells with nanomolar concentrations of the Hsp90 activity inhibitors (17AAG, NVP-AUY922, geldanamycin and radicicol) caused a marked delay in the post-radiation formation and subsequent disappearance of the γH2AX foci (sites of double-strand DNA breaks/repair [30]) in the cell nuclei (Fig 7A). The non-pretreated cells exhibited the maximal nuclear γH2AX foci at 1 h and 3 h after irradiation followed by a gradual decrease in the amounts of foci per nucleus at 6 h and 24 h, which appears to reflect the unaffected DNA damage response and a normal dynamics of DNA break repair. In contrast, the Hsp90 inhibitor-pretreated cells had a very low accumulation of γH2AX foci in the nuclei at 1 h and 3 h after irradiation; then their nuclear foci reached a peak at 6 h, and later, at 24 h after radiation exposure, the intensity of γH2AX foci still remained near the maximum (Fig 7A). Apparently, such γH2AX foci dynamics indicates the compromised DNA damage response and DNA break repair. The same effect of the Hsp90 inhibitors, namely the delay in post-radiation formation/disappearance of γH2AX foci, took place in other cell cultures which became radiosensitized as a result of the inhibitory pretreatments such as HeLa, KTC-1, PC-3, Myc-CaP and HT 1080 cancer cells, or actively proliferating vascular endothelial cells. In contrast, no delay in the dynamics of post-radiation formation/disappearance of nuclear γH2AX foci was observed in the Hsp90 inhibitor-pretreated cell cultures whose radiosensitivity was not increased by such pretreatments: HBL-100, FRO and B16 tumor cells, non-cancerous BJ and 293 cells (Fig 7B) or quiescent endothelial cells (not shown). This difference may explain the selectivity in the Hsp90 inhibitor-induced radiosensitization because the abundance of γH2AX foci in the cell nuclei at the later time point (24 h) after irradiation suggests the uncompleted DNA break repair, and such inability of the irradiated cell to timely accomplish the repair of double-strand DNA breaks can lead to its death [30,38].

**Fig 8:**
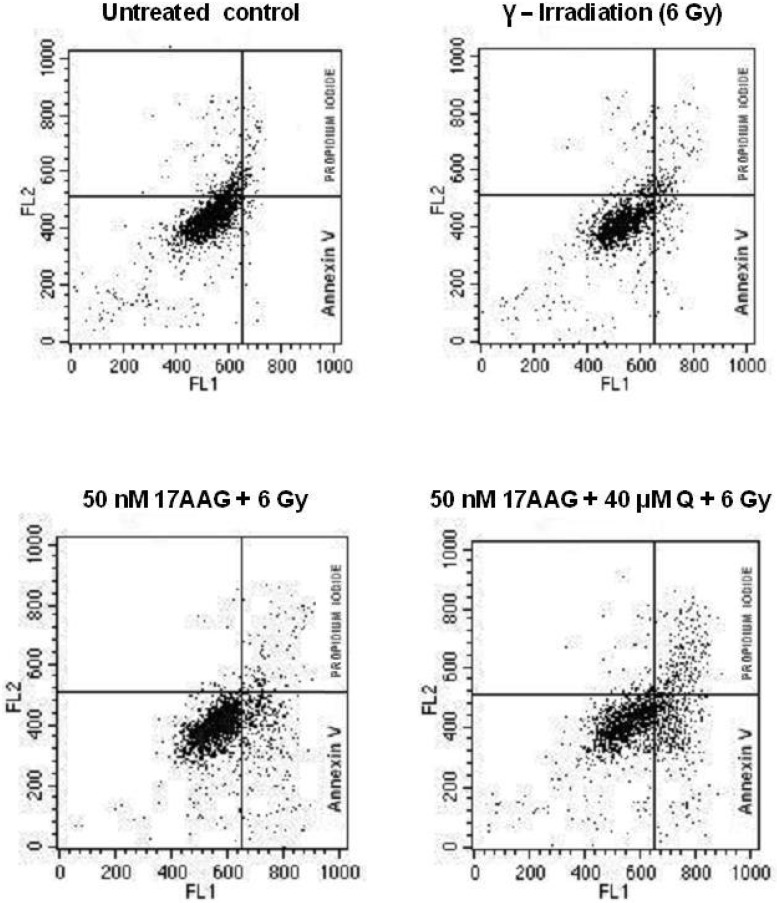
Targeting the Hsp70 induction in Hsp90 inhibitor-treated cancer cells enhances their apoptotic death following radiation exposure. MCF-7 breast cancer cells were either untreated (control) or exposed to γ-photons (6 Gy) without any drug pretreatment or after 24 h incubation with 50 nM 17AAG alone or in combination with 40 μM quercetin (**Q**). After 48 h, the cells were stained with FITC-annexin V/propidium iodide (PI) and analyzed by flow cytometry. The presented distribution of stained cell subpopulations demonstrates the considerable enhancement of post-radiation apoptosis (FITC-annexin V-positive, PI-negative cells) and secondary necrosis (PI-positive cells) in samples where the 17AAG-induced up-regulation of Hsp70 was fully blocked by quercetin (see Table 3). Analogous effects were also observed on HeLa, PC-3 and Myc-CaP cancer cells and actively proliferating vascular endothelial cells (not shown).

Finally, we made an attempt to establish a cause of the enhancement of radiosensitizing effects of combinations of the Hsp90 activity inhibitors with inhibitors of the Hsp70 induction. We found that the double-inhibitor co-treatments did not increase amounts of the nuclear γH2AX foci in irradiated MCF-7, HeLa, KTC-1, PC-3, Myc-CaP and HT 1080 cancer cells and did not delay a disappearance of the formed foci in them as compared with the action of Hsp90 inhibitors alone (not shown). This indicates that the prevention of the Hsp70 induction does not enhance the radiation-induced breakage of nuclear double-strand DNA and does not retard the post-radiation DNA break repair. However, we observed the significant enhancement of the post-radiation cell death, in particular, apoptosis in MCF-7 breast cancer cells irradiated after the double-inhibitor treatments (Fig 8, Table 3). Some part of those apoptotic cells later became PI-positive (so called “secondary” necrosis), the summarized values of total cell death (apoptosis + necrosis) were substantially higher in the samples of cancer cells co-treated with the two inhibitors and γ-radiation. Similar observations were also made on HeLa, KTC-1, PC-3, Myc-CaP and HT 1080 cancer cells and the actively proliferating endothelial cells (not shown). These findings indicate that the drug-induced prevention of Hsp70 induction/up-regulation in Hsp90 inhibitor-sensitive cells can intensify their apoptotic/necrotic death following radiation exposure rather than affect their DNA damage/repair.

**Table 3:** Effects of the Hsp90 activity inhibition with 17AAG and suppression of the Hsp70 induction with quercetin (Querc) on post-radiation apoptosis and necrosis in MCF-7 breast cancer cells.

Cell death		50 nM 17AAG + 40 μM Querc	γ-Irradiation (6 Gy)	50 nM 17AAG + 6 Gy	50 nM 17AAG + 40 μM Querc + 6 Gy
**% Apoptosis**	**24 hours**	4.23 ± 0.54	9.06 ± 1.03	16.56 ± 2.35*	25.78 ± 2.93**
	**48 hours**	5.52 ± 0.71	10.78 ± 1.36	18.28 ± 2.13*	28.62 ± 3.05**
**% Necrosis**	**24 hours**	3.01 ± 0.04	6.23 ± 0.75	11.14 ± 1.42*	15.25 ± 1.76*
	**48 hours**	4.47 ± 0.05	7.81 ± 0.9	14.31 ± 1.58*	18.84 ± 1.91*

The presented data are average percentages ± SEM of apoptosis (PI-negative, FITC-annexin V-stained cells) or necrosis (PI-positive cells) at 24 and 48 hours after γ-irradiation (6 Gy).

*—significant difference from the respective effects of γ-irradiation (6 Gy) alone, p < 0.05;

**—significant difference from the respective values marked with *, p< 0.05, and from the respective effects of γ-irradiation alone, p < 0.01.

## Discussion

Our data show that: (1) clinically achievable (nanomolar) concentrations of the Hsp90 activity inhibitors cause up-regulation of Hsp70 in some cancer cell lines and actively proliferating cell cultures of the vascular endothelium, whereas in other cancer and normal cells such response is lacking; (2) only cell cultures with early and prominent Hsp70 induction after treatments with the Hsp90 inhibitors become radiosensitized by such treatments; (3) the levels of Hsp70 induction in cells treated with the Hsp90 inhibitors positively correlate with a degree of the radiosensitization; (4) artificial (drug-induced) prevention of the Hsp70 up-regulation in cells treated with Hsp90-inhibiting radiosensitizers can significantly enhance the radiosensitizing effect toward those cells.

Induction of Hsp70 in mammalian cells treated with the Hsp90 activity inhibitors was described many years ago [20]. The molecular mechanism of this phenomenon is usually explained by suppression of the chaperone function of Hsp90 that leads to activation of HSF1 triggering expression of the *heat shock*-genes that, in turn, results in transient up-regulation of inducible Hsps (first of all, Hsp70) in the target cells. It seems intriguing why the Hsp70 induction does not occur equally in all cell cultures pre-incubated with the cell-permeable Hsp90 inhibitors? This cannot be explained by different regulation of the HSF1-mediated stress response because no diversity in the Hsp70 expression was observed after thermal stressing of all cell cultures studied (see Fig 1 and Table 1).

As for non-cancerous cell lines 293 and BJ, it was possible to cause the marked Hsp70 induction in them if higher (1.5–2 μM) concentrations of the Hsp90 inhibitors were used (see Fig 2); however, it seems important that clinically achievable (nanomolar: 35–500 nM) concentrations of those inhibitors induced Hsp70 only in some cancer cell lines and in actively proliferating endothelial cells. The discrepancy between the effects in cancerous and non-cancerous cells may be due to the certain (hyper-activated) state of a major pool of Hsp90 in cancer cells that implies a certain molecular conformation whose affinity to Hsp90 inhibitors like 17AAG is more than 100-fold higher as compared with that of “latent” Hsp90 in normal (non-cancerous) cells [39]. If so, much lower concentrations of the inhibitors are required for inactivation of cytosolic Hsp90 (and, respectively, for the HSF1 activation/Hsp70 induction) in malignant cells than in normal ones. Such a suggestion is supported by our results presented in Fig 6. It seems likely that in the *in vivo* situation, nanomolar concentrations of the Hsp90-inhibiting radiosensitizers will act selectively on the target tumor without aggravation of radiation damage to adjacent normal tissues.

Likewise, we suppose that the same mechanism may define the diversity in cellular responses between actively proliferating and quiescent endothelial cells. If the actively proliferating endothelial cells resemble to some extent cancer cells, their Hsp90 may similarly be in the activated state with certain conformation and higher affinity to Hsp90-binding drugs than that in the quiescent vascular endothelium. In such a case, the Hsp90-inhibiting radiosensitizers will preferentially sensitize proliferating endothelial cells involved in tumor-stimulated angiogenesis that can additionally enhance the beneficial action of radiotherapy.

It seems also interesting why some cancer cell lines remain in sensitive to treatments with nanomolar concentrations of the Hsp90 inhibitors? Comparing results obtained on MCF-7 and MCF-7/MDR1 cells, it seems likely that in some cases the absence or strong attenuation of tumor cell responses to the Hsp90 inhibitors can be due to overexpression and high activity of the membrane P-glycoprotein that takes place in malignancies with multidrug resistance. In our model, after pre-incubation with verapamil (a blocker of the drug-pumping-out activity of P-glycoprotein), HBL-100, FRO and B16 tumor cell lines still did not react to treatments with the Hsp90 inhibitors; thus, probably, other (non-related to MDR1/P-glycoprotein) mechanisms are involved. While molecular machinery of Hsp90-dependent intracellular signal transduction can vary in different tumors, it is possible that in some cancer cell lines almost all cytosolic Hsp90 is in inside tight multicomponent protein complexes with its co-factors and client proteins so that the sensitive (ATP-ase) domain of the chaperone becomes unavailable for molecules of the inhibitor caught in the cytoplasm. In any event, there are tumors and cancer cell lines which can somehow be resistant to the cytotoxic and radiosensitizing effect of the Hsp90 inhibitors; consequently, there is the great need in a biomarker which would enable to predict responses of target cells to the inhibitory treatment.

Based on our findings, we believe that the early and prominent Hsp70 induction (or its absence) may be such a predictive marker. In fact, if the newly expressed inducible form of Hsp70 became abundant in Hsp90 inhibitor-treated cells, this clearly indicates that the drug has penetrated into these cells and suppressed the cytosolic Hsp90 chaperone activity in them. As functioning of this 90 kDa chaperone is necessary for the radioresistance of cancer cells [9–15], the Hsp70 induction in them can be considered as an indicator of the chaperone dysfunction and a predictor of the radiosensitization. On the contrary, the absence of Hsp70 induction in the Hsp90 inhibitor-treated cells will mean that their Hsp90 is not inactivated and Hsp90-dependent radioprotective mechanisms still function and, therefore, no radiosensitization will be achieved. This is exactly what is seen in Fig 7A and 7B where the different effects of 17AAG on the post-radiation DNA break repair are demonstrated in two cell cultures inducing Hsp70 (MCF-7 cells) or non-inducing Hsp70 (293 cells) in response to the drug treatment. The fact that 17AAG, NVP-AUY922, geldanamycin and radicicol did not affect the post-radiation formation/disappearance of nuclear γH2AX foci in HBL-100, FRO, B16, 293 cells (Fig 7B) and quiescent endothelial cells means that the machinery of repair of double-strand DNA breaks in those cells really is insensitive to the Hsp90 activity inhibitors. In the other group of cell cultures (MCF-7, HeLa, KTC-1, PC-3, Myc-CaP, HT 1080 and actively proliferating vascular endothelial cells), the same Hsp90 inhibitors first delayed the irradiation-responsive formation of nuclear γH2AX foci and then prevented their disappearance (see Fig 7A for example); this indicates the drug-provoked failure of the DNA break-repairing cellular pathways and a susceptibility of these cells to the Hsp90-inactivating radiosensitizers. Since an accumulation of nuclear γH2AX foci reflects the phosphorylation of histone H2AX as a result of activation of ataxia-telangiectasia mutated (ATM) and that this is one of primary and characteristic radioprotective reactions aimed at forming the functional sites of repair of the double-strand DNA breaks [30,38], it is possible to explain the apparent lag in the post-radiation formation of nuclear γH2AX foci in 17AAG-pretreated MCF-7 cells (see Fig 7A) by the transient delay in the Hsp90-dependent ATM activation. Other researchers previously reported that Hsp90 inhibitor DMAG impairs the radiation-induced activation of ATM in tumor-derived cell lines MiaPaCa [33], NCI-H460, and A549 [34], which resulted in interfering with the post-radiation DNA repair and radiosensitizing the DMAG-treated cells. It seems likely that similar ATM-involving mechanisms were responsible for the delay in formation of nuclear γH2AX foci in MCF-7, HeLa, KTC-1, PC-3, Myc-CaP, HT 1080 and actively proliferating vascular endothelial cells irradiated after treatments with the Hsp90 inhibitors. As for the marked delay in the post-radiation disappearance of nuclear γH2AX foci in cells radiosensitized by the Hsp90 inhibition (see Fig 7A for example), this suggests a lethal protraction in the nuclear DNA break repair and may be due to inactivation or degradation of some Hsp90-dependent components of cellular DNA-repairing pathways. Actually, multiple components of the double-strand DNA break repair machinery, including BRCA1 and BRCA2, DNA-PKcs, MRE11/RAD50/NBN complex, CHK1, FANCA and others, have been identified as client proteins of the Hsp90 chaperone (see for a review [37]); thus, their dysfunction resulted from the Hsp90 inactivation may lead to the inability of Hsp90 inhibitor-pretreated cells to survive consequences of the DNA-breaking radiation exposure.

It should be noted that comparative evaluation of the treatment-responsive expression of inducible Hsp70 in cells treated with the Hsp90 inhibitors will allow: (1) to predict an ability (or inability) of the radiosensitization of target cells by this inhibitor, (2) to suggest the effective concentrations of the Hsp90 inhibitor(s) upon which the radiosensitizing effect can be expected, (3) to know whether this radiosensitization will be selective toward tumor cells. Furtherore, the antibodies with specificity to an inducible form of Hsp70 are commercially available, while techniques of immunodetection of Hsp70 in bio-samples are relatively simple and do not require long-term experiments or expensive equipment. We hope that such immunodetection of inducible Hsp70 as the predictive marker will be applicable for freshly harvested biopsy samples and tissue sections, and in the nearest future, we plan to examine this on histological preparations from tumors grown in mice.

Inducible Hsp70 is not the sole cellular protein whose expression is altered in response to the drug-provoked Hsp90 dysfunction. The intracellular levels of some other Hsps (e.g. Hsp27, Hsp40 etc) may also augment after the action of Hsp90 inhibitors [20] but we found only slight increase in Hsp27 and Hsp40 in the treated cells (not shown) that was incomparable with the potent up-regulation of Hsp70. Moreover, certain malignant growth-related proteins (e.g. Raf-1, ErbB2, Akt, HIF-1α, and others) are clients of the Hsp90 chaperone and may undergo proteasomal degradation in cancer cells treated with the Hsp90 activity inhibitors [6,8,10]; if so, the expression levels of these proteins may become down-regulated and such signs may be detectable as indicators of the Hsp90 dysfunction. However, we believe that the expression of inducible Hsp70 is, in all respects, the more sensitive and convenient biomarker because its treatment-responsive alteration (several-fold up-regulation), if occurs, is usually more dramatic and can be immunodetected earlier and easier than gradual degradation of any Hsp90 client proteins. Furthermore, the Hsp90 inhibitor-responsive activation (phosphorylation) of HSF1 may also indicate the dysfunction of Hsp90 but it does not seem to be a good immunodetectable biomarker due to a transient sojourn of HSF1 in its active (phosphorylated) form and the labile (phosphatase-sensitive) epitope. According to our unpublished observations, the powerful induction of Hsp70 and the radiosensitization do not always follow the transient HSF1 activation in mammalian cells treated with the Hsp90 inhibitors, thus the up-regulation of inducible Hsp70 seems to be the better predictive marker.

Importantly, the radiosensitization of tumor cell by the Hsp90 activity inhibitors can be significantly enhanced by preventing the induction/up-regulation of Hsp70 that is possible to render with known inhibitors of HSF1. Schilling et al. [40], using NZ28, already reported the effectiveness of such an approach for radiosensitization of several tumor cell lines and their data are in full consent with our findings obtained with NZ28 and the other HSF1 inhibitors (quercetin, KNK437, triptolide) in other cell lines. According to our observations, prevention of the Hsp70 induction in the Hsp90 inhibitor-treated cancer cells results in their more intensive apoptosis following ү-photon exposure (see Fig 8 and Table 3). The anti-apoptotic activity of inducible Hsp70 is well known and this cytoprotective chaperone has multiple targets for interrupting apoptotic signal transduction pathways (reviewed in [41]), so it seems logical that the drug-induced blockage of the treatment-responsive Hsp70 up-regulation in target cells promotes apoptosis. We have found that such radiosensitization enhanced by preventing the Hsp70 induction/accumulation was not accompanied by aggravation in the nuclear double-strand DNA breakage after irradiation (not shown). However, Gabai et al. [23] had previously demonstrated that depletion of inducible Hsp70 constitutively expressed in tumor cells enhances formation of nuclear γH2AX foci after irradiation that proves a contribution of the inducible 70-kDa chaperone to protection of nuclear DNA from genotoxic exposures. In our conditions, treatments with inhibitors of the HSF1-mediated Hsp70 induction were short term and did not decrease the basal levels of constitutive Hsp70 expression (Fig 5A), therefore, we did not see the enhancing effect on H2AX foci. In turn, our findings suggest that the Hsp90 inhibition-mediated increase in Hsp70 over its basal level confers neither additional protection of nuclear DNA nor its accelerated repair from radiation-induced breakage but, instead, confers cytoprotection against post-radiation apoptosis. We suppose that after treatments with the Hsp90 inhibitors, up-regulated Hsp70 enables irradiated cells to avoid apoptosis otherwise being quite likely during the protracted DNA repair. Therefore, prevention of the Hsp70 up-regulation in Hsp90 inhibitor-treated cells will enhance their post-radiation death via apoptosis triggered as a result of the incomplete repair of DNA damages.

Taken together these experimental data support the idea to enhance the cancer cell radiosensitization by simultaneous inhibiting both the Hsp90 activity and the associated Hsp70 induction. We suppose that the double drug combination *Hsp90 activity inhibitor + Hsp induction inhibitor* may help to overcome the high radioresistance of some human tumors and sensitize them to radiotherapy. Although the effective (micromolar) concentrations of quercetin, KNK437 and NZ28 seem too high for clinical application, the potent and selective enhancement of cancer cell radiosensitization conferred by those inhibitors suggests a feasibility of the proposed approach. As for triptolide, this drug exerted the strong effects even being used in extremely low (3–5 nM or even lesser) concentrations (see Figs 4 and 5 and Table 2) which are clinically achievable, although its toxicity to normal cells/tissues should be also evaluated. Probably, the combination *Hsp90 inhibitor + triptolide* should be tested in various models of anticancer therapy. We would like to notice that, while many small molecule-based inhibitors of the Hsp90 activity currently undergo preclinical and clinical trials as potential antitumor or tumor-sensitizing agents, there is a great need in development of pharmacological blockers of the undesirable HSF1 activation/Hsp induction in the target tumors. Joint administration of such blockers with the Hsp90 activity-inhibiting radiosensitizers would yield more effective elimination of malignant cells and cells of growing tumor vasculature; consequently, this would enhance the tumor radiation response and improve the outcome of radiotherapy.
